# Body Mass Index and Mortality in a Middle-aged Japanese Cohort

**DOI:** 10.2188/jea.15.70

**Published:** 2005-06-01

**Authors:** Rumiko Hayashi, Motoki Iwasaki, Tetsuya Otani, Naren Wang, Hiroko Miyazaki, Sasazawa Yoshiaki, Shigenobu Aoki, Hiroshi Koyama, Shosuke Suzuki

**Affiliations:** 1 Department of Virology and Preventive Medicine, Gunma University School of Medicine.; 2 Epidemiology and Prevention Division, Research Center for Cancer Prevention and Screening, National Cancer Center.; 3 Department of Public Health, Gunma University School of Medicine.; 4 Npo International Eco-Health Research Group.; 5 Faculty of Social and Information Studies, Gunma University.

**Keywords:** Body Mass Index, Japan, Mortality, Obesity, Prospective Studies

## Abstract

BACKGROUND: The relative risk of mortality in low and high body mass index (BMI) categories in various ethnic groups remains a controversial subject.

METHODS: To examine the relationship between BMI and mortality, a population-based prospective cohort study was conducted in two areas of Gunma Prefecture, Japan, in 1993. A total of 5,554 men and 5,827 women aged 40-69 years completed a self-administered questionnaire and were followed up until the year 2000. The hazard ratios (HRs) were estimated by the Cox proportional hazards model for different BMI classes.

RESULTS: During the seven year follow-up period, 329 men and 147 women died. As compared with those in the reference BMI category (22.0-24.9 kg/m^2^), men and women in the lowest BMI category (<18.5 kg/m^2^) had a HR (95% confidence interval [CI]) of death from all-causes of 2.66 (1.59-4.46) and 3.14 (1.38-7.13), respectively, and women in the highest BMI category (28.0+ kg/m^2^) had a HR of death of 3.25 (1.48-7.15), after adjusting for all possible confounding factors including smoking and after excluding deaths occurring during the first three years of follow-up.

CONCLUSION: In this prospective study of a Japanese cohort consisting of subjects ranging in age from 40 to 69 years, the curve depicting the relationship between BMI and all-cause mortality was L-shaped in men and U-shaped in women.

The relative risks of mortality in subjects with low and high body mass index (BMI) have been assessed in many large prospective studies, and it has been reported that the relationship between BMI and mortality can be represented by a J-, L-, or U-shaped curve, or is linear,^1^^-^^12^ although the interpretation still remains under debate. In western countries, higher BMIs have been shown to be associated with a higher mortality.^1^^-^^4^^,^^9^^-^^11^ However, most studies conducted in Japan have not indicated any increase in mortality with increasing BMI, especially in men,^5^^,^^6^^,^^12^ and have suggested that, on the contrary, leanness may be a greater health hazard in this population.

While the National Health and Nutrition Surveys (NHANES｣: 1988-94) in the United States reported a prevalence of obesity (BMI=30.0) of 19.9% among men and 24.9% among women,^13^ in Japan, the prevalence of obesity in adults aged 15 years or older was estimated in 1993 to be only 1.6% among men and 2.2% among women.^14^ Although the prevalence of overweight subjects (BMI=25.0) was quite low in Japan as compared with that in the United States,^13^ it has recently increased rapidly in this country, especially in men.^15^ The consequences of this increase in the prevalence of overweight subjects are expected to become serious in the future, because obesity is known to be associated with cardiovascular diseases.

The purpose of this study was to clarify the health risks of being overweight as well as those associated with being underweight, based on the survival status during a seven-year follow-up period in a Japanese cohort. We conducted a baseline questionnaire survey of residents aged 40-69 years in Komochi Village and Isesaki City, Gunma Prefecture, Japan, in 1993.

## METHODS

### Study cohort

The design of this cohort study, called the Komo-Ise study, has been described previously.^16^^,^^17^ Briefly, the subjects consisted of 4,875 persons from Komochi Village and 7,755 persons from the downtown district of Isesaki City, consisting of all residents aged 40 to 69 years, identified in the Basic Resident Resisters of Komochi Village as of September 1992, and of Isesaki City as of August1993. Both the districts are in Gunma prefecture, 100 km north of Tokyo. The village had a population of 12,141, with 3,284 households, and the city had a population of 120,236, with 40,335 households, according to the ‘95 census.^18^

Self-administered questionnaires were distributed through the respective municipal government offices to all of the residents of Komochi Village in January 1993 and to the residents of the downtown area of Isesaki City in October 1993, and completed questionnaires in sealed envelopes were collected by the officers after a few weeks. A total of 11,565 subjects from both areas returned their responses to the questionnaire (response rate: 91.6%) in the baseline survey. Non-respondents were not re-contacted. Ohta et al.^16^ have reported that the non-response bias and selection bias were negligible in this baseline study. The questionnaire items included questions on the physical status, such as the body weight and height, demographic variables such as age and education, lifestyle variables, including smoking, drinking and exercise habits, and the Todai Health Index (THI), which is a check list of physical and mental complaints.^19^^,^^20^

The Komo-Ise study was approved by the Ethical Review Committee of Gunma University, Gunma, Japan.

### Body Mass Index

The BMI (weight [kg]/height [m]^2^) of the subjects was calculated from the self-reported weight and height. Kawada et al. evaluated the validity of using the self-reported body weight and body height in this cohort for the assessment.^21^ The weight and height data from health check-ups were available for 1,823 individuals. Pearson’s correlation coefficient between the self-reported and measured values was 0.97 for body weight, 0.97 for body height, and 0.94 for BMI. As for the systematic error in the case of predicting measured values from self-reported values, the slope of body weight, body height, and BMI in the regression lines were 1.02, 0.96 and 0.98, and the intercept of body weight, body height, and BMI in the regression lines were -0.96, 5.57 and 0.54, respectively, thus, indicating the validity of using the self-reported weight and height in the baseline questionnaire. In our analysis, the BMI values were classified into five categories: less than 18.5, 18.5 to 21.9, 22.0 to 24.9, 25.0 to 27.9, and 28.0 or more.

### Other variables

Cigarette smoking habit was assessed by using the question, “Do you smoke cigarettes?”, with the answer options being “Never”, “Past”, or “Current”. Current smokers were also asked regarding the number of cigarettes they smoked per day. Drinking habit was evaluated by asking the question, “Do you drink a lot of alcoholic beverages?”, with the answer options of “Yes”, “A little”, or “Hardly ever or Never”. Physical activity was estimated by asking the question, “Do you exercise regularly?”, with the answer options being “Often”, “Sometimes”, or “Never”. The sociodemographic factors enquired about were the sex, age, and educational background.

### Follow-up

We followed up the survival status of all the subjects from 1993 through 2000. Deaths and migrations were identified in the Basic Residents Registers of each area, and the dates of deaths and migrations were recorded. Deaths, which accounted for 488 cases, were identified by the death certificates at the public health center in each area, with the permission of the Management and Coordination Agency, the Government of Japan. According to the International Classification of Diseases, Tenth Revision (ICD-10), the causes of death were coded as follows: deaths from all cancers (ICD-10: C00-C97), all circulatory system diseases (ICD-10: I00-I99), ischemic heart diseases (ICD-10: I20-I25), cerebrovascular diseases (ICD-10: I60-I69), and external causes (ICD-10: V01-Y89). The observation period for six men and women could not be confirmed in spite of a careful follow-up survey. We excluded subjects who gave incomplete information in respect of the body weight and/or height (n=178). The remaining 5,554 men and 5,827 women, including 476 deaths (329 men and 147 women), were included in this analysis. There were 385 subjects (3.4% of the analytic cohort) who migrated from the study areas and were lost to follow-up.

### Statistical Analyses

For each subject, the person-years of follow-up were counted from January 31, 1993, and October 31, 1993, for the subjects in Komochi and Isesaki, respectively, until March 31, 2000, the date of death, or the date of migration away from the study areas, whichever occurred first.

The Cox proportional-hazards model was used to examine the relation between BMI and all-cause mortality and cause-specific mortality using SPSS^®^, version 10.0J, for Windows. We calculated the hazard ratio (HR) of death for the five categories of BMI (reference 22.0-24.9), after adjustment for age in 1993, study area (Komochi, Isesaki), cigarette smoking status (never smokers, past smokers, current smokers smoking 1-19 cigarettes per day, and current smokers smoking at least 20 cigarettes per day), alcohol drinking habit (never, light drinkers, and heavy drinkers), physical activity (never, often or sometimes), and level of education (junior high school or less, high school, and college or more). The age of the subjects in 1993 was fed into the models as a continuous variable, and other variables were categorized using dummy variables. Further, we analyzed the association between BMI and all-cause mortality after stratifying the subjects by the smoking status, to avoid the confounding effect of smoking. All of the analyses were performed independently for men and women.

Prior health status was thought to have the largest effect during the early follow-up period, and those already ill at the time of the baseline study probably had a higher risk of death. Thus, in order to reduce the possible effect of unreported or unrecognized health conditions at the baseline on the mortality, cases dying within the first three years of follow-up were excluded from the analyses.

The 95% confidence interval (CI) was calculated for the HRs. All the P values were two-tailed.

## RESULTS

### Baseline Characteristics by BMI Category

The overall mean BMI values for the 5,554 men and 5,827 women were 23.0 kg/m^2^ (standard deviation=2.8) and 23.0 kg/m^2^ (standard deviation=3.1), respectively. The mean BMI and the smoking rate of the respondents were almost the same as those reported from the National Nutrition Survey, Japan (1993).^14^ The baseline characteristics of the study subjects classified by the five categories of BMI are given in Table 1 by sex. The proportion of subjects in the lowest BMI category (<18.5) was 4.2% for men and 5.3% for women, and the prevalence of overweight subjects (BMI=25.0) was 22.0% in men and 23.2% in women. The mean age tended to be higher in the lower BMI categories, especially in men. The prevalence of cigarette smoking was inversely associated with the BMI. The prevalence of subjects who did not drink alcohol was smallest for the BMI category of 22.0-24.9, while the prevalence of subjects who engaged in regular physical activity was largest for the BMI category of 22.0-24.9. The level of education tended to be higher in the higher BMI categories in men, but in the lower BMI categories in women.

**Table 1.** Baseline characteristics by category of body mass index.

	Body Mass Index (kg/m^2^)				

	-18.4	18.5-21.9	22.0-24.9	25.0-27.9	28.0+
	Men				
No. of subjects*	234 (4.2)	1749 (31.5)	2345 (42.2)	1002 (18.0)	224 (4.0)

Mean age (years)	58.0	54.0	54.0	53.1	52.2
Mean weight (kg)	46.9	55.7	63.5	71.4	80.5
Mean height (cm)	164	164	165	165	164
Mean BMI (kg/m^2^)	17.5	20.6	23.4	26.2	29.7

Cigarette smoking (%)					
Never	22	20	28	32	27
Past	15	14	19	18	16
1-19 cigarettes/day	23	21	15	12	9
20≦ cigarettes/day	40	46	38	38	48

Alcohol drinking (%)					
Never	36	22	20	22	33
Light	46	52	53	51	44
Heavy	19	26	28	28	23

Physical activity (%)					
Never	68	57	51	55	53
Often, sometimes	32	43	49	45	47

Level of education (%)					
Junior high school or less	52	45	40	40	44
High school	35	37	38	38	36
College or more	13	18	22	22	21

	Women				
No. of subjects*	311 (5.3)	1954 (33.5)	2213 (38.0)	977 (16.8)	372 (6.4)
					
Mean age (years)	56.1	53.1	54.7	55.9	55.6
Mean weight (kg)	40.8	48.2	54.3	60.7	68.3
Mean height (cm)	153	153	152	152	151
Mean BMI (kg/m^2^)	17.4	20.6	23.4	26.2	29.9

Cigarette smoking (%)					
Never	77	86	87	88	81
Past	2	2	2	2	4
1-19 cigarettes/day	12	8	6	7	7
20≦ cigarettes/day	9	5	5	4	8

Alcohol drinking (%)					
Never	64	56	55	62	64
Light	33	41	42	36	33
Heavy	3	2	3	3	3

Physical activity (%)					
Never	64	59	58	62	66
Often, sometimes	36	41	43	38	34

Level of education (%)					
Junior high school or less	40	37	46	54	54
High school	41	45	38	33	33
College or more	19	19	16	13	13

*: Percentages in parentheses.

As for the confounding effects of cigarette smoking, alcohol drinking, physical activity and level of education in the evaluation of the BMI-mortality association, these variables were also included in the model, in addition to the study area and age.

### Mortality by the BMI Category

As of March 31, 2000, the cohort members had contributed 35,709 and 37,921 person-years of observation (an average of 6.4 and 6.5 years of follow-up per subject; figures for men and women, respectively). During the follow-up, a total of 329 men and 147 women died. Thus, more than twice as many men as women died. The causes of death in the men were as follows: 145 (44%) deaths were due to cancer (37, lung cancer; 26, stomach cancer; 15, liver cancer) and 99 (30%) deaths were due to all circulatory system diseases (38, cerebrovascular disease; 29, ischemic heart disease). The causes of death in the women were as follows: 58 (39%) deaths were due to cancer (10, breast cancer; 7, stomach cancer; 7, pancreatic cancer) and 47 (32%) deaths were due to all circulatory system diseases (24, cerebrovascular disease; 7, ischemic heart disease). In regard to other internal causes of death in the cohort during the study period (19% [n=63] in men and 22% [n=32] in women of the total number of deaths), 15 men and 4 women died of pneumonia, and 8 men and 3 women died of liver cirrhosis. The number of deaths from external causes was 22 (7%) and 10 (7%) in men and women, respectively.

The person-year of follow-up, number of deaths and the HRs of death with a 95% CI are shown in Table 2 by sex, according to the five BMI classes. There were statistically significant elevations in the all-cause mortality risk in lean men and women and in obese women after adjustment for age, study area, cigarette smoking, alcohol drinking, physical activity, and level of education. As compared with that in the reference BMI group (22.0-24.9 kg/m^2^), the HRs (95%CI) were 2.59 (1.74-3.85) and 2.93 (1.62-5.30) for men and women, respectively, in the lowest BMI category (<18.5 kg/m^2^), and 2.71 (1.51-4.88) for women in the highest BMI category (28.0+ kg/m^2^). Although a statistically significant elevation in risk was not observed for men in the highest BMI category, the HR was of borderline significance (HR [95%CI]=1.63 [0.93-2.87]). The elevated risk of deaths among men and women in the lowest BMI category and women in the highest BMI category remained unchanged even after excluding deaths occurring in the first three years of follow-up.

**Table 2.** Hazard ratios (HRs) of all-cause and cause-specific mortality by body mass index.

	Body Mass Index (kg/m^2^)				

	-18.4	18.5-21.9	22.0-24.9	25.0-27.9	28.0+
	Men				
All-cause mortality					
Person-year of follow-up	1409	11292	15095	6472	1441
No. of deaths	41	111	116	46	15
Multivariate HR*	2.59 (1.74-3.85)	1.25 (0.94-1.66)	1.00 (reference)	1.06 (0.74-1.53)	1.63 (0.93-2.87)
Multivariate HR^†^	2.66 (1.59-4.46)	1.33 (0.93-1.91)	1.00 (reference)	1.21 (0.78-1.90)	1.18 (0.51-2.74)

Cancer mortality					
No. of deaths	16	56	49	18	6
Multivariate HR*	1.93 (1.01-3.68)	1.37 (0.92-2.05)	1.00 (reference)	0.89 (0.51-1.58)	1.59 (0.67-3.73)

Circulatory system disease mortality					
No. of deaths	8	31	38	17	5
Multivariate HR*	2.17 (0.93-5.06)	1.27 (0.73-2.19)	1.00 (reference)	1.47 (0.77-2.78)	1.88 (0.65-5.42)

Other internal causes mortality					
No. of deaths	12	20	20	8	3
Multivariate HR*	3.70 (1.67-8.20)	1.17 (0.60-2.28)	1.00 (reference)	1.03 (0.45-2.38)	1.57 (0.46-5.38)

	Women				
All-cause mortality					
Person-year of follow-up	1968	12681	14488	6394	2390
No. of deaths	18	43	41	24	21
Multivariate HR*	2.93 (1.62-5.30)	1.49 (0.94-2.35)	1.00 (reference)	1.34 (0.78-2.31)	2.71 (1.51-4.88)
Multivariate HR^†^	3.14 (1.38-7.13)	1.98 (1.07-3.65)	1.00 (reference)	1.92 (0.96-3.84)	3.25 (1.48-7.15)

Cancer mortality					
No. of deaths	7	13	17	12	9
Multivariate HR*	2.65 (1.07-6.61)	0.98 (0.46-2.10)	1.00 (reference)	1.45 (0.65-3.24)	2.59 (1.05-6.40)

Circulatory system disease mortality					
No. of deaths	4	17	14	5	7
Multivariate HR*	2.56 (0.79-8.31)	2.14 (0.97-4.73)	1.00 (reference)	1.09 (0.37-3.20)	3.26 (1.17-9.08)

Other internal causes mortality					
No. of deaths	6	10	6	6	4
Multivariate HR*	5.12 (1.45-18.1)	2.29 (0.76-6.84)	1.00 (reference)	2.51 (0.76-8.23)	3.17 (0.75-13.4)

* : Adjusted for age in 1993 (continuous), study area (Komochi, Isesaki), cigarette smoking (never smokers, past smokers, current smokers smoking 1-19 cigarettes per day, current smokers smoking at least 20 cigarettes per day), alcohol drinking (never drinkers, light drinkers, heavy drinkers), physical activity (never, often or sometimes), and level of education (junior high school or less, high school, college or more).

† : Examined with the exclusion of deaths occurring during the first three years of follow-up.

95% confidence intervals in parentheses.

Table 2 also shows the relation between BMI and mortality from cancer, circulatory system diseases, and other internal causes of deaths. The risk of cancer mortality showed a similar trend to the all-cause mortality in both men and women. In relation to deaths from circulatory system diseases, a significantly elevated mortality was observed among women with a BMI of 28.0+ kg/m^2^. The risk of other internal deaths showed a statistically significant increase in both men and women with a BMI of <18.5 kg/m^2^. Because the number of deaths was limited, the relation between BMI and mortality from external causes could not be examined.

The HRs were calculated for different categories of smoking separately, in order to examine whether smoking modified the relation between BMI and mortality (Table 3). The pattern of elevated risks of death in males of all categories of smoking from the lowest BMI category (<18.5 kg/m^2^) showed a similar trend to the all-cause mortality. Also, in current male smokers, a statistically significant elevation of the HR was observed in the highest BMI category (28.0+ kg/m^2^). In female never-smokers, a statistically significant elevation of the HR was observed in the lowest and highest BMI categories.

**Table 3.** Hazard ratios (HRs) of all-cause mortality by smoking status and body mass index.

	Body Mass Index (kg/m^2^)				

	-18.4	18.5-21.9	22.0-24.9	25.0-27.9	28.0+
	Men				
Never smokers					
Person-year of follow-up	294	2211	4107	1995	395
No. of deaths	9	11	24	17	3
Multivariate HR*	3.74 (1.71-8.17)	0.81 (0.39-1.67)	1.00 (reference)	1.20 (0.62-2.32)	0.92 (0.21-3.91)

Past smokers					
Person-year of follow-up	177	1432	2768	1161	232
No. of deaths	10	24	20	4	1
Multivariate HR*	4.04 (1.69-9.66)	2.05 (1.09-3.83)	1.00 (reference)	0.57 (0.19-1.69)	0.51 (0.07-3.85)

Current smokers					
Person-year of follow-up	849	7254	7739	3088	794
No. of deaths	19	72	64	24	11
Multivariate HR*	1.88 (1.08-3.26)	1.15 (0.80-1.65)	1.00 (reference)	1.12 (0.69-1.83)	2.45 (1.28-4.71)

	Women				
Never smokers					
Person-year of follow-up	1473	10511	12327	5460	1866
No. of deaths	14	29	32	18	11
Multivariate HR*	3.39 (1.78-6.45)	1.24 (0.74-2.07)	1.00 (reference)	1.03 (0.55-1.91)	2.05 (1.00-4.20)

* : Adjusted for age in 1993 (continuous), study area (Komochi, Isesaki), alcohol drinking (never drinkers, light drinkers, heavy drinkers), physical activity (never, often or sometimes), and level of education (junior high school or less, high school, college or more).

95% confidence intervals in parentheses.

## DISCUSSION

The authors conducted a population-based cohort study to assess the relation between BMI and mortality in Japan, where the distributions of the relative body weight and mortality patterns are different from those in western countries. The curve depicting the relationship between BMI and all-cause mortality in this study was L-shaped in men and U-shaped in women, after adjustment for possible confounders, including the smoking status and other factors.

Many previous studies evaluating the relationship between the body weight and mortality have had important methodological limitations, including failure to control for confounding by cigarette smoking and failure to consider the effects of subclinical disease on weight.^22^ To avoid these confounding effects, we examined the BMI-mortality relation for different smoking categories separately, and excluded deaths that occurred within the first three years of the follow-up period from this analysis. Despite these steps, our findings remained unchanged. Further, we also analyzed the association between BMI and mortality after adjusting for occupation, in addition to for the level of education, to avoid the confounding effects of socioeconomic status. We found that the association between BMI and mortality persisted even after adjusting for occupation (data not shown). Women tended to have a higher mortality risk due to being overweight than men.

Lee et al.^10^ reported a J-shaped relation between BMI and mortality, and concluded that there was no evidence of excess increased mortality risk among lean men. On the other hand, some researchers have reported that leanness does increase the risk of mortality.^1^^,^^2^^,^^4^^-^^9^^,^^12^ For example, Tsugane et al. examined the BMI-mortality association in a study of 40,000 Japanese men and women followed up for 10 years and showed that leanness increased the mortality risk in both men and women.^7^ In this study also, the leanest group (BMI<18.5 kg/m^2^), both among men and women, was shown to have a higher risk of death, even after exclusion of early deaths and adjusting for or stratifying the smoking status vs. the risk of death. The results suggested that being underweight by itself was a risk factor for mortality in the Japanese population. Malnutrition is known to reduce immunity and impair resistance to infection.^23^ Shirasaki^24^ has argued that leanness may diminish the resistance to every disease.

The prevalence of overweight subjects (BMI=25.0+ kg/m^2^) in the Japanese population has been small as compared with that in western countries. However, it recently increased rapidly in Japan, with the trend being similar to that seen in western countries. Between 1976-80 and 1988-94, the prevalence of overweight subjects (BMI=25.0+ kg/m^2^) increased from 46% to 54% in the United States.^13^ On the other hand, the prevalence of overweight subjects (BMI=25.0+ kg/m^2^) in Japanese men aged 40-49 years increased from 24% in 1981 to 32% in 2001. Marked increase in the prevalence of overweight subjects was also found in the following groups: men aged 50-59 years (20% to 32%), men aged 60-69 years (20% to 31%), women aged 60-69 years (28% to 31%). However, the prevalence of overweight subjects has not shown any increase over the last 20 years in Japanese women aged 40-59 years.^15^ The health hazards of the overweight state and obesity are well known in western countries, making the populations in these countries more suited to examination of the relationship between high BMI values and mortality than our study population. Although few studies in Japan have reported an increased risk of mortality in overweight subjects, there was a clearly elevated mortality risk in women in the highest BMI category in this study. For men, the HR was of borderline significance, although no statistically significant elevation of the risk was observed in association with higher BMI values.

The observed sex differences in the BMI-mortality associations may reflect the influences of body fat distribution and muscular development. Bigaard et al.^25^ reported that both high body fat and low fat-free mass are independent predictors of the overall mortality. It has also been hypothesized that higher mortality among those with a low BMI is the result of low fat-free mass rather than low fat mass.^26^ In general, for the same value of BMI, women tend to have a higher proportion of body fat than men. This might contribute to the increased mortality risk at higher values of BMI and the remarkably elevated HR in the lowest BMI group in women. In future research, measurements of the body composition and fat distribution would be needed to examine the association between adiposity and mortality.

The cause of death modified the relation between BMI and mortality in both men and women. The increased risk of death from cancer and internal deaths other than cancer and circulatory diseases at low BMI levels suggests the effect of a low BMI on the risk of death in this study. Lower BMI values have been reported to be associated with an increased risk of some cancers, including cancer of the lung, which is the most frequent cause of cancer death in Japan, although higher BMI values have also been associated with an increased risk of some cancers.^27^ The relation between a high BMI and mortality from cardiovascular disease has been well established.^1^^-^^5^^,^^8^^-^^11^ On the other hand, some reports indicate that leanness may be associated with an increased risk of cerebrovascular disease.^28^^,^^29^ In the current study, the other internal causes of death included pneumonia (15 men and 4 women). Baumgartner et al.^26^ suggested increased susceptibility to infectious diseases among the very lean. Because the observation period in the present study was only seven years and the number of deaths was limited, a longer period may be required before any conclusions can be drawn on this analysis for specific causes.

The response rate of 91.6% in this study was rather high. As reported previously by Ohta et al.^16^, the selection bias seemed to be negligible.

Our study, however, had several limitations. The baseline data did not include objective data, such as physical and laboratory data, except for some subjects in this study. Furthermore, there was the insufficient number of subjects with a BMI of over 30.0 kg/m^2^ in our cohort (1.3% among men and 2.2% among women), for us to reasonably evaluate the effect of severe obesity. Despite these limitations, our findings suggest that a low BMI in both men and women, and a high BMI in women are associated with an increased risk of death.

Our results highlight the serious health risk associated with leanness in the Japanese population, reported from several Japanese-population-based studies.^5^^-^^7^^,^^12^ Although not emphasized for the Japanese population so far, in this study, the results indicated the impact of being overweight on health, just as in western countries. The prevalence of overweight subjects (BMI=25.0+ kg/m^2^) has recently increased rapidly in Japan, especially in men; thus, being overweight could be expected to become a serious public health concern in the future. In summary, both the underweight and overweight states are important determinants of premature death in the Japanese population.
